# Supplementary material for: Assessment of radio-activation using spectroscopy in medical linear accelerators
Source: PLoS One. 2024 Apr 18;19(4):e0301435. doi: 10.1371/journal.pone.0301435 (PMC11025788; doi:10.1371/journal.pone.0301435)
Supplement: Attachment — Submitted filename: Response to Reviewers.docx [file pone.0301435.s002.docx]

**Response to Reviewers**

2023.12.19

A. **Journal Requirements:**
1. Please ensure that your manuscript meets PLOS ONE's style requirements, including those for file naming. The PLOS ONE style templates can be found at

https://journals.plos.org/plosone/s/file?id=wjVg/PLOSOne_formatting_sample_main_body.pdf and https://journals.plos.org/plosone/s/file?id=ba62/PLOSOne_formatting_sample_title_authors_affiliations.pdf

→ Thank you for your guidance. In response to your comments, we have revised the manuscript to comply with the PLOS ONE style requirements. The revised files have been divided into two parts: one for the Title & Affiliation and the other for the Abstract & Main body. We appreciate your support in this process and look forward to any further comments you may have.

2. In your Methods section, please provide additional information regarding the permits you obtained for the work. Please ensure you have included the full name of the authority that approved the field site access and, if no permits were required, a brief statement explaining why.

→ Thank you for your question. The equipment used in this study was radiotherapy devices used for cancer treatment. These facilities have already received usage approval from the regulatory authorities. Therefore, there was no need for additional permits for this study. We appreciate your attention to detail and look forward to any further comments you may have.

3. Thank you for stating the following in the Acknowledgments Section of your manuscript:
   “This research was supported by the National Research Council of Science & Technology grant by the Korea government (Ministry of Science and ICT) (No.CAP22041-000) & National Research Foundation of Korea (NRF) grant funded by the Korean government (No. RS-2022-00144201) & the Nuclear Safety Research Program through the Korea Foundation of Nuclear Safety (KoFONS) using the financial resource granted by the Nuclear Safety and Security Commission (NSSC) of the Republic of Korea (No.2205013).”

We note that you have provided funding information that is not currently declared in your Funding Statement. However, funding information should not appear in the Acknowledgments section or other areas of your manuscript. We will only publish funding information present in the Funding Statement section of the online submission form. 
Please remove any funding-related text from the manuscript and let us know how you would like to update your Funding Statement. Currently, your Funding Statement reads as follows: 
“The author(s) received no specific funding for this work.”

Please include your amended statements within your cover letter; we will change the online submission form on your behalf.

→ Thank you for your question. Following your advice, we have revised the Acknowledgments section and added a Funding section to our manuscript. The amended content is as follows:

- Acknowledgments

We would like to express our heartfelt thanks to all those who have helped and supported with this article. Our deepest gratitude goes to Jihyun Yu and Byungchae Lee from the R&D Institute at Sae-An Enertech Corp., whose invaluable contributions to data analysis and insightful feedback have been indispensable.

- Funding

This research was supported by the National Research Council of Science and Technology (NST) grant by the Korea government (MSIT) (No. CAP22041-000) & the National Research Foundation of Korea (NRF) grant funded by the Korean government (RS-2022-00144201, NRF-2020M2D9A3094170) & Nuclear Safety Research Program through the Korea Foundation of Nuclear Safety (KoFONS) using the financial resource granted by the Nuclear Safety and Security Commission (NSSC) of the Republic of Korea (No. 2205013).

4. In your Data Availability statement, you have not specified where the minimal data set underlying the results described in your manuscript can be found. PLOS defines a study's minimal data set as the underlying data used to reach the conclusions drawn in the manuscript and any additional data required to replicate the reported study findings in their entirety. All PLOS journals require that the minimal data set be made fully available. For more information about our data policy, please see <http://journals.plos.org/plosone/s/data-availability.>

Upon re-submitting your revised manuscript, please upload your study’s minimal underlying data set as either Supporting Information files or to a stable, public repository and include the relevant URLs, DOIs, or accession numbers within your revised cover letter. For a list of acceptable repositories, please see <http://journals.plos.org/plosone/s/data-availability#loc-recommended-repositories.> Any potentially identifying patient information must be fully anonymized.

Important: If there are ethical or legal restrictions to sharing your data publicly, please explain these restrictions in detail. Please see our guidelines for more information on what we consider unacceptable restrictions to publicly sharing data: <http://journals.plos.org/plosone/s/data-availability#loc-unacceptable-data-access-restrictions.> Note that it is not acceptable for the authors to be the sole named individuals responsible for ensuring data access.

We will update your Data Availability statement to reflect the information you provide in your cover letter.

→ Thank you for your additional information. In accordance with the guidelines provided by the PLOS ONE journal, we will submit the minimal data set underlying the results described in our manuscript as Supplementary materials. This data set includes all the necessary information to reach the conclusions drawn in the manuscript and to replicate the reported study findings in their entirety. Specifically, the Supplementary materials contain the raw data of the measured spectrum, including energy information for each channel and detected nuclide information. We appreciate your guidance in this matter.

In page 5, line 131-132:

“We have organized the cps and isotopic information by energy into a table, which is included in S1 Table.”

**Additional Editor Comments:**

Q1) Title = not ok. It didn't reflect all the written tittle especially the “implication for rad.....”. Please revise.

→ Thank you for your comment. We revised the title as follow.

“Assessment of Radio-Activation Using Spectroscopy in Medical Linear Accelerators”

Q2) Abstract - Reader would like to know in first 3-4 lines the objectives, statement of problems or maybe the application that best suited this Zr-Bi-Mn-borate glass. No conclusion has been drawn in the abstract.

→ Thank you for your comment. In accordance with your suggestion, we have revised the content of the 3-4th lines of the abstract to include the relevant details. We appreciate your insightful feedback. The amended content is as follows:

“In radiotherapy, when photon energy exceeding 8 MV is utilized, photoneutrons can activate the components within the gantry of the linear accelerator (linac). At the end of the linac's lifecycle, radiation workers are tasked with its dismantling and disposal, potentially exposing them to unintentional radiation. This study aims to identify and measure the radioisotopes generated by this activation through spectroscopy, and to evaluate the effective dose rate.”

Q3) Abstract, line 5 - We chose nine medical linacs based on.......

→ We changed the sentences as follow your comment. The text now reads, “We chose nine medical linacs based on different conditions such as …”.

Q4) Line 3 - TomoTheraphy? Or just tomotheraphy with small letter?

→ Dear Reviewer, thank you for your comment. Regarding your question about the term “Tomotherapy”, we have revised it to be in lowercase as “tomotherapy”.

Q5) Line 5 - remove “Typically, 6, 10, and 15 MV energies are used during photon beam therapy using a linac for treatment”

→ Dear Reviewer, thank you for your insightful comment. In line with your suggestion, we have removed the sentence “Typically, 6, 10, and 15 MV energies are used during photon beam therapy using a linac for treatment”.

Q6) Line 4 - 103 all in South Korea? Need rot spoecify.

→ Dear Reviewer, thank you for pointing out the need for clarification. We have revised the sentence for clarity. It now reads, “According to data from the Radiation Safety Information System, as of 2023, 103 medical institutions in South Korea use radiation generators.” We appreciate your helpful feedback.

Q7) Line 6-7 - Revise with full stop. Please consider active voice without the bracket.

→ Certainly, here's the revised sentence in active voice and without brackets:

“The number of linacs in South Korea with maximum MV energy are 64 for 6 MV, 104 for 10 MV, and 73 for 15 MV.”

Q8) Line 8-9 - Photon-induced radionuclides? Photon-induced activity phenomenon?

→ Dear Reviewer, thank you for your suggestion. I have revised the sentence to more accurately describe the 'radioactive phenomenon'. It now reads:

“When an energy of 8 MV or more is used, various parts located in the head of the linac are continuously irradiated, leading to a phenomenon known as photon-induced activity.”

Q9) Line 11-12 - “that emits gamma rays” and exposes the worker/patient with unnecessary few hundred nGy/hr of gamma radiation dose? Need to elaborate the consequence of photon-induced activity phenomenon.
    → Dear Reviewer, thank you for your insightful comment. Indeed, the consequence of the photon-induced activity phenomenon is crucial. In relation to your question, we have addressed the impact and result of unnecessary exposure on line 19, which states:

“Radiation workers are inevitably exposed to radiation when removing equipment from its treatment room after the beam is shut down.”

This elaboration is intended to convey the potential risks involved for the workers. We appreciate your valuable guidance.

Q10) Line 36 -37 - Clarify your aims and objective of the work. the sentence “by assessing the activation phenomenon doesn’t reflecting the work procedure of identifying photon-induced radionuclides and measuring gamma dose rates.

→ Dear Reviewer, your suggestion is appreciated. I'd like to revise the first sentence of the statement to clarify the aims and objectives of the study. It would now read:

“In this study, our objective was to assess the impact of activation in linacs through the analysis of radionuclides and measurement of gamma dose rates.”

I believe this revision aligns with your feedback. Please let me know if there are any other aspects you'd like me to address.

Q11) Line 77 - spectra were measured measured or analysed??

→ Dear Reviewer, thank you for your question. In this context, I used the term “measured” because the spectra themselves are acquired or collected, rather than analyzed. I appreciate your attention to detail and please feel free to share any more questions or comments you might have.

Q12) Methodology - please elaborate the HPGe detector calibration in details for both energy and efficiency calibration. It is inapproriate to use only one source of energy calibration from Co-60. If it is sufficient to use only Co-60 then explain why? Because of 2 gamma peak; 1.17 MeV and 1.3 MeV?

→ Dear Reviewer, thank you for raising this important point.

In terms of the efficiency calibration for each energy, we utilized efficiency values derived from a certified reference material (CRM) of ^152^Eu prior to measurement. Furthermore, a ^60^Co source was employed for re-verification during the in-situ process. We selected the ^60^Co source specifically due to its ability to confirm the gamma peaks at 1.17 and 1.3 MeV.

We have integrated the aforementioned explanations into the main body of the paper.

“For energy & efficiency calibration, we utilized values based on the certified reference material of ^152^Eu, keeping in mind the potential inaccuracies due to the variance in density, shape, and size of the iron-based radioactive waste generated from the linac. Prior to spectra acquisition, we used a ^60^Co authoritative source for in-situ re-checking of the energy-channel correlation, leveraging the identified gamma peaks at 1.17 and 1.3 MeV.”

Q13) Dose rate measurement - please explain the type of survey meter? Scintillation detector NaI for gamma dose rate? Or gas-filled for alpha beta detection? Please provide the image of in-situ measurement. What its the distance of measurement from sources?

→ According to the comment from the reviewer, I have written as follows, and I have added the measurement photo as Figure 2.

“The dose rate was measured at the beam exit window using a survey meter (ESM FH 40 GL; Thermo Inc., Germany), which is a gamma meter with an internal proportional counter tube. The measurement of the dose rate with the survey meter was conducted at a distance of 0 cm from the front of the beam exit window. (Fig 2)”

**Fig 2.** Set-up of HPGe detector for spectroscopic measurements

Q14) Results and discussion - nuclide identification and dose rate need to be compared with other works. Please add a table and do more comparison.

→ Thank you for your comment. In accordance with your suggestion, we have added a table comparing our measurement results with those of previous studies (Table 4). Also, we have made some modifications to the main text as follows:

“We compared the results of our study with those of previous research [3, 4, 7]. Despite the generational evolution of the equipment, the nuclides detected across different linac models exhibited minimal disparities. Contrary to prior studies, our evaluation was based on actual treatment conditions and usage, without any intentional irradiation. Consequently, our results did not identify nuclides with extremely short half-lives, such as ^62^Cu (half-life 9.74 min) and ^28^Al (half-life 2.24 min).

Both our measurements and the outcomes of preceding studies predominantly detected nuclides with half-lives under 100 days. Four nuclides with half-lives exceeding 100 days (^54^Mn, ^57^Co, ^60^Co, ^65^Zn) were commonly identified, irrespective of the manufacturer. As for the nuclide ^181^W, it mainly possesses a low energy peak under 100 keV, which appears to be excluded in the study by Fisher et al.”

**Table 4.** Comparison of Nuclide Detection between Our Study and Previous Studies

| Manufacturer | | Elekta | | Siemens | | Varian | |
| --- | --- | --- | --- | --- | --- | --- | --- |
| Nuclide | Half-life | Our data | Fisher et al [4] | Our data | Fisher et al [4] | Our data | Fisher et al [3,4] Fujibuchi et al [7] |
| ^24^Na | 15.02 h | D | D | D | D | D | D |
| ^28^Al | 2.24 m |  | D |  | D |  | D |
| ^51^Cr | 27.70 d |  | D |  |  |  |  |
| ^54^Mn | 312.2 d | D | D | D | D | D | D |
| ^56^Mn | 2.579 h | D | D | D | D | D | D |
| ^57^Co | 270.9 d | D | D | D | D | D | D |
| ^57^Ni | 35.65 h | D | D | D | D | D | D |
| ^58^Co | 70.80 d | D | D | D | D | D | D |
| ^59^Fe | 44.63 d | D | D | D | D |  | D |
| ^60^Co | 5.271 y | D | D | D | D | D | D |
| ^62^Cu | 9.74 m |  | D |  | D |  | D |
| ^64^Cu | 12.70 h | D | D | D | D | D | D |
| ^65^Zn | 243.7 d | D | D | D | D | D | D |
| ^82^Br | 35.30 h | D | D | D | D | D | D |
| ^99^Mo | 66.02 h | D | D | D | D |  |  |
| ^116m^In | 54.3 m |  |  |  |  |  | D |
| ^122^Sb | 2.700 d | D | D | D | D | D | D |
| ^124^Sb | 60.21 d | D | D | D | D | D | D |
| ^181^W | 121.2 d | D |  | D |  | D | D |
| ^184^Re | 38.00 d | D | D |  |  |  |  |
| ^187^W | 23.83 h | D | D | D | D | D | D |
| ^196^Au | 6.183 d | D | D | D | D | D | D |
| ^198^Au | 64.80 d | D |  | D |  | D | D |
| ^203^Pb | 52.93 h | D |  |  |  | D |  |

Q15) Fig. 2 - not clear, please provide big scale and good resolution.

→ Thank you for your suggestion. We have revised Fig. 3 according to your feedback. The revised figure has been enlarged for better clarity and understanding. We appreciate your input and hope that the updated figure meets your expectations.

Q16) Linear accelerator no. - please change to no. of reference no alphabets.

→ In response to your comment, we have changed the linear accelerator numbers from alphabets to numeric digits 1-9.”

Q17) Line 126 - 136 - please include “gamma” to indicate the gamma dose rate.

→ Thank you for your comment. As per your suggestion, I have included the term “gamma” in the text to specify the gamma dose rate.

Q18) Fig.4 - provide big scale of figure.

→ Thank you for your suggestion. We have made revisions to Fig.4 according to your feedback. The revised figure has been enlarged for better clarity and understanding. We appreciate your input and hope that the updated figure meets your expectations.

Q19) Line 170 - how do you calculate the annual exposure dose? provide the Equation or appropriate dose conversion factor from any study or from Monte Carlo simulation. From what distance? Is this annual effective dose?

→ Thank you for your question. Based on the gamma dose rate measurements, a simple calculation was performed. We considered the dose rate per hour for 8 hours a day, 5 days a week, and 50 weeks in a year to calculate the possible annual dose.

“According to the measurement results for the 15 MV equipment, if work starts immediately after the patient’s treatment is finished, the exposure is 2.4 mSv annually. *(1.2 µSv/h×8 hours×5 days×50 weeks =2.4 mSv per year)* However, if work starts after 1.5 h the exposure is 0.54 mSv per year. *(0.268 µSv/h×8 hours×5 days×50 weeks =0.54 mSv per year)*”

Q20) Line 185 - 191 and Line 197 - 201 - redundant. It has been elaborated in intro.

→ Thank you for your comment.

The content on lines 185-191 pertains to the management of radioactive waste components generated during the disposal of linear accelerators, as per the new regulations established in 2023. Following your suggestion, we have removed any overlapping content with the Introduction.

Similarly, we have eliminated any redundancy with the Introduction in lines 197-201. Your feedback is greatly appreciated, and if there's anything else you'd like to discuss, please let me know.

Q21) Line 210 - justify why it is needed to identify the usage history.......? Why?

→ Thank you for your comment. I apologize if the term “identify” caused any confusion. As explained in the text, we found that the usage conditions of radiation therapy equipment (energy and workload) affect the level of radioactivity. The more the equipment is used at 10 MV, the more patients it treats, and the higher the weekly operation load, the higher the level of radioactivity. This also affects the time it takes to reduce the gamma dose during the disposal process.

Therefore, we proposed checking the usage history of the equipment in advance when planning for disposal. To avoid confusion, I have replaced the term “identify” with “confirm”. I hope this clarification addresses your query.

Q22) Conclusion - please write a new conclusion to conclude. Don't summarise the whole work.

→ Thank you for your feedback. Given your suggestion, I propose the following revised conclusion:

“In conclusion, our study offers pivotal insights into the radio-activation of linacs operating at energies exceeding 10 MV, and provides practical guidelines for their safe disposal. Our findings underscore the significance of considering the energy usage, ratio, and operating load in evaluating radioactivity levels. Although the workload does influence dose rate and nuclide outcomes, we have found that the primary energy used and its ratio exert a more substantial impact on radio-activation. There were no discernible differences attributable to the equipment manufacturers. We recommend a waiting period following the cessation of the beam, especially for equipment operating at 15 MV, and a conservative approach for linacs with energies below 10 MV. Our results contribute not only to the understanding of radio-activation but also lay the groundwork for establishing safety management standards for the disposal and relocation of therapeutic linacs.”

This conclusion highlights the main findings without summarizing the entire work, and it emphasizes the potential impact and future implications of the study. Please let me know if you have any further suggestions or comments.

**Reviewer #1&2 Comments**

1. Is the manuscript technically sound, and do the data support the conclusions?

The manuscript must describe a technically sound piece of scientific research with data that supports the conclusions. Experiments must have been conducted rigorously, with appropriate controls, replication, and sample sizes. The conclusions must be drawn appropriately based on the data presented.

Reviewer #1: Yes

Reviewer #2: Yes

2. Has the statistical analysis been performed appropriately and rigorously?

Reviewer #1: Yes

Reviewer #2: Yes

3. Have the authors made all data underlying the findings in their manuscript fully available?

The [PLOS Data policy](http://www.plosone.org/static/policies.action#sharing) requires authors to make all data underlying the findings described in their manuscript fully available without restriction, with rare exception (please refer to the Data Availability Statement in the manuscript PDF file). The data should be provided as part of the manuscript or its supporting information, or deposited to a public repository. For example, in addition to summary statistics, the data points behind means, medians and variance measures should be available. If there are restrictions on publicly sharing data—e.g. participant privacy or use of data from a third party—those must be specified.

Reviewer #1: Yes

Reviewer #2: Yes

4. Is the manuscript presented in an intelligible fashion and written in standard English?

PLOS ONE does not copyedit accepted manuscripts, so the language in submitted articles must be clear, correct, and unambiguous. Any typographical or grammatical errors should be corrected at revision, so please note any specific errors here.

Reviewer #1: Yes

Reviewer #2: Yes

5. Review Comments to the Author

Please use the space provided to explain your answers to the questions above. You may also include additional comments for the author, including concerns about dual publication, research ethics, or publication ethics. (Please upload your review as an attachment if it exceeds 20,000 characters)

Reviewer #1: The author has provided a wonderful study about the radiation dose rate at the exit window. It would be interesting if the author has data set not only at the exit window but around the head of the gantry as well. Did they perform the survey meter only at the exit window or for the sorrounding gantry as well?.

→ Thank you for your comment. In our study, we focused on measurements at the beam exit window. We assumed that sufficient shielding would be in place around the gantry, hence we did not perform measurements there. We appreciate your suggestion and will consider expanding our measurement areas in future studies. If there's anything else you'd like to discuss, please let me know.

Reviewer #2: The study is relevant because the standards for handling radioactive materials in Korea are unclear.
The proposed method will be potentially useful as a general basis for establishing standards for linac radioactivity assessment and safety management

→ Thank you for your insightful comment. We agree that the standards for handling radioactive materials in Korea need clarity. We hope that our study can contribute to the development of a more comprehensive standard for linac radioactivity assessment and safety management. Your recognition of the relevance and potential impact of our work is greatly appreciated. If there's anything else you'd like to discuss, please let me know.
