# Supplementary material for: Assessment of radio-activation using spectroscopy in medical linear accelerators
Source: PLoS One. 2024 Apr 18;19(4):e0301435. doi: 10.1371/journal.pone.0301435 (PMC11025788; doi:10.1371/journal.pone.0301435)
Supplement: Attachment — Submitted filename: Response to Reviewers_240306.docx [file pone.0301435.s003.docx]

**Response to Reviewers**

2024.03.06

A. **Academic Editor comments:**
1. Line 133: The FH 40 G-L energy detection range spans from 36 keV to 1.3 MeV. Technically, gamma emitters above 1.3 MeV, such as Sb, Mn, Br, Ni, etc., are not being measured. Therefore, the results of the measured gamma dose rates in Fig. 4 might be underestimated. Additionally, the author did not address the beta contribution. If beta is not considered in this work for occupational exposure, please highlight or make assumptions regarding why beta is considered negligible or low energy. Since the measurements were taken at 0 cm from the source, high-energy beta might contribute in FH 49 G-L, maybe? Furthermore, no other details are provided for the ESM 40 G-L (energy linearity, performance calibration, alpha-beta discriminator).

→ Thank you for your valuable feedback. In accordance with your suggestion, we conducted a re-measurement using an FH 40 G-L 10 survey meter, which has an energy detection range of 30 keV to 4.4 MeV. We have updated Figure 4 in the manuscript accordingly.

**(Page 5, Line 123-126)**

The dose rate was measured at the beam exit window using a survey meter (Thermo FH 40 G-L 10; Thermo Inc., Germany). This gamma meter has an internal proportional counter tube. We used a dose-rate calibration factor of 1.1, as provided by the Korea Research Institute of Standards and Science (KRISS), the National Metrology Institute.

Fig 4. Results of dose rate measurements in front of beam exit window

Additionally, according to the paper by Fisher H W et al. (ref [3]), the most significant reaction within the energy range covered by medical accelerators is the nuclear photo effect. The neutrons produced by this reaction can undergo their own nuclear reactions, primarily neutron capture. Given this physical background of activation, we focused on gamma emissions, excluding beta contribution. We also recommend using the survey meter, which is the most commonly used detector in the Department of Radiation Oncology. Survey meters are designed for dose-rate measurements and are only calibrated for dose-rate due to calibration with a single energy source, so they do not consider energy linearity or alpha-beta discrimination.

We appreciate your insightful comments which enhance the quality of our research. If you have any further comments, please do not hesitate to let us know.

2. Table 3: Are the results for Canberra or Ortec? Please combine the table to present a comparison, as this work assesses qualitative rather than quantitative aspects.

→ Table 3 presents results obtained using both Canberra and Ortec HPGe detector. Each detector was used for spectroscopy with a different linac, and this point has been clarified in the main text. Additionally, we added information on the HPGe detector used for spectroscopy for each linac in Table 1. In response to your feedback, we have combined Tables 3 and 4 for a more comprehensive comparison.

**(Page 5, Line 113-114)**

We utilized both Canberra and Ortec systems for measurements, each employed for spectroscopy of a different linac of the facility.

3. TABLE 4: Instead of marking "D" in the table, provide the true detected energies. The title of the table must start with a lowercase letter.

→ Thank you for your comment. We have added the key energy for each nuclide to Table 3. However, since the detected energy varies for each nuclide, we have only listed the key gamma energy that serves as the guideline for determining the presence of a nuclide in the table. Additionally, we have added the following content to the main text. (We deleted the table 4.)

4. Unit of uSv/h: Please revise. Do not use italics. Use Times New Roman. Additionally, the R^2 should be in italics.

→ We appreciate your feedback. We have revised the document in line with your comments.

5. Line 229-231: Please do not write linear equations and text in the body of the article. Use symbols 'X' and '='. Do not italicize the text. How do you consider 8 hours of exposure? Please justify, and if there is solid evidence of workers sitting close to the gantry head, provide references or any reports.

→ Thank you for your feedback. We have revised the text according to your suggestions and have included Equation 1 and related content in the manuscript.

**(Page 10, Line 213-215, 220)**

We calculated the annual Dose equivalent (H) based on Equation (1), where D is the measured dose rate per hour, and T is the working time. The working time (T) was set considering the standard working hours of workers: 8 hours per day, 5 days per week, and 50 weeks per year.

H (mSv/y) = D (uSv/u) * T (8 hours * 5 days * 50 weeks) - Equation (1)

Considering a typical radiation therapy setting, radiation therapists nearly work near the gantry head when they lay patients on the couch, set up for treatment, install the electron block, or assist the patient after treatment. Also, when linear accelerators are being decommissioned or disassembled for transfer and installation at other facilities, workers perform the disassembly work near the linear accelerator gantry head. We made this evaluation with these work environments in mind and aimed for a conservative assessment.

6. Text style: Use "Justify." Maintain the same font size. Ensure that affiliations are presented in the same size.

→ We appreciate your feedback. We have revised the document in line with your comments.
